# The Macroeconomic Impact of Increasing Investments in Malaria Control in 26 High Malaria Burden Countries: An Application of the Updated EPIC Model

**DOI:** 10.34172/ijhpm.2023.7132

**Published:** 2023-10-04

**Authors:** Edith Patouillard, Seoni Han, Jeremy Lauer, Mara Barschkett, Jean-Louis Arcand

**Affiliations:** ^1^Department of Health Financing and Economics, World Health Organization, Geneva, Switzerland; ^2^Korea Institute for International Economic Policy, Sejong, Korea; ^3^Strathclyde Business School, University of Strathclyde, Glasgow, UK; ^4^Federal Institute for Population Research and Department of Public Economics, German Institute of Economic Research (DIW Berlin), Berlin, Germany; ^5^Global Development Network, New Delhi, India; ^6^Mohammed VI Polytechnic University, Rabat, Morocco; ^7^Foundation for Studies and Research on International Development (FERDI), Clermont Ferrand, France

**Keywords:** Investment Case, Economic Evaluation, Malaria Control

## Abstract

**Background:** Malaria remains a major public health problem. While globally malaria mortality affects predominantly young children, clinical malaria affects all age groups throughout life. Malaria not only threatens health but also child education and adult productivity while burdening government budgets and economic development. Increased investments in malaria control can contribute to reduce this burden but have an opportunity cost for the economy. Quantifying the net economic value of investing in malaria can encourage political and financial commitment.

**Methods:** We adapted an existing macroeconomic model to simulate the effects of reducing malaria on the gross domestic product (GDP) of 26 high burden countries while accounting for the opportunity costs of increased investments in malaria. We compared two scenarios differing in their level of malaria investment and associated burden reduction: sustaining malaria control at 2015 intervention coverage levels, time at which coverage levels reached their historic peak and scaling-up coverage to reach the 2030 global burden reduction targets. We incorporated the effects that reduced malaria in children and young adolescents may have on the productivity of working adults and on the future size of the labour force augmented by educational returns, skills, and experience. We calibrated the model using estimates from linked epidemiologic and costing models on these same scenarios and from published country-specific macroeconomic data.

**Results:** Scaling-up malaria control could produce a dividend of US$ 152 billion in the modelled countries, equivalent to 0.17% of total GDP projected over the study period across the 26 countries. Assuming a larger share of malaria investments is paid out from domestic savings, the dividend would be smaller but still significant, ranging between 0.10% and 0.14% of total projected GDP. Annual GDP gains were estimated to increase over time. Lower income and higher burden countries would experience higher gains.

**Conclusion:** Intensified malaria control can produce a multiplied return despite the opportunity cost of greater investments.

## Background

**Key Messages**

**Implications for policy makers**

- There is ample evidence on the economic benefits of malaria control. The potential net gains in projected gross domestic product (GDP) from reduced malaria burden on one hand and increased investments that divert resources from other important areas on the other hand have not been previously estimated.
- Under a range of assumptions, this study shows that intensified malaria control could produce a multiplied net return in terms of economic growth.
- The study used a macroeconomic model called Economic Projections of Illness and Costs (EPIC) that can be easily adapted to different diseases and conditions and calibrated to different countries to support the development of investment cases in health.
- EPIC can complement other types of tools to inform priority-setting in health.

**Implications for the public**

After remarkable success in the fight against malaria, investments in malaria control and disease burden reductions have plateaued. Malaria continues to have a devastating impact on population lives and livelihoods: deaths, notably in young children, reduce the total size of the future workforce, while infections throughout life reduce productivity because of work and school absenteeism. Intensifying malaria control requires substantial resources, which, on one hand, averts infections and treatment costs while, on the other hand, reduces investment opportunities in other important areas. Quantifying the net economic return of investing in malaria can better reflect the wide impact of the disease and provide useful information for decision makers.

In 2015, the global malaria community celebrated the achievements of halting and reversing the global malaria incidence.^1,2^ A more than two-fold increase in funding between 2000 and 2015 had permitted the expansion of key malaria control interventions, with this scale-up contributing to dramatically reduce the burden of malaria.^3^ Since this remarkable period, the level of investments and the disease burden have remained virtually unchanged.^4,5^ Each year less than half of the investments needed to reach the global 2030 burden reduction targets are invested and more than 240 000 million cases and 590 000 related deaths are reported.^4^ Malaria is also closely associated with other health conditions, such as anaemia and cognitive deficits.^6-9^

 Globally, malaria mortality affects predominantly young children while clinical malaria affects all age groups throughout life.^8,10^ The resulting deaths and illnesses have been reported to have significant economic consequences now and for the future.^11^ At a micro level, malaria expenditures burden households and governments with most of primary healthcare spending for malaria paid out from domestic sources.^12,13^ At household level, malaria reduces labour participation and productivity because of work absenteeism due to adults own sickness or time spent caring for a child.^14-16^ Malaria also reduces older children and young adolescents’ educational attainments and future adult employment.^17-19^ Like other diseases, malaria affects economic progress through lost capital and future income. Financial and physical capital may be depleted through reduced savings, dis-savings, the sale of household assets and livestock and/or borrowing.^20,21^ At business level, a decrease in labour participation can reduce the production of firms if the productive contribution of workers cannot be compensated by other production factors or if absent workers cannot be replaced by new workers with sufficient skills and experience.^22,23^ At a macro level, malaria mortality can reduce the total size of the labour force while aggregated morbidity may reduce the total size and productivity of the workforce, human capital accumulation, and ultimately national economic output.^16^ Malaria also absorbs a significant amount of domestic resources for prevention and treatment,^24^ diverting part of these resources from other productive investments, notably infrastructure, equipment and machinery among others, which can ultimately also impact aggregate economic output.

 Quantifying the economic burden of malaria and the return of investing in malaria control can encourage political and financial commitment.^25^ The inclusion of impacts beyond health can thus better reflect the wide impact of a disease such as malaria and can help raise awareness among policy-makers of the implications of these economic consequences for national economic progress.^25^

 There is ample literature on the economic burden and benefits of malaria control, especially in terms of the micro- and macro-economic effects of changes in morbidity and mortality, treatment cost and productivity. Yet limited consideration has been given to the effects of changes in savings that can hamper economy-wide physical capital accumulation and as a result economic outcomes. Many studies have used the cost-of-illness (COI) approach to estimate the direct and indirect costs of malaria including treatment costs and losses in labour force participation and associated income.^26-31^ The underlying assumption is that the estimated economic value in COI studies represents the potential benefits of malaria control and elimination if it had been implemented.^32^ Several other studies use econometric methods such as cross-country growth regression, quasi-experiments studies or macroeconomic models to estimate the impact of malaria on aggregate economic outcomes.^16,19,33-37^ For example, these studies may consider the relationship between the gross domestic product (GDP) and malaria incidence or/and between the growth of industries with the same share of labor intensity and malaria incidence,^16,33,34^ or the effects of changes in economic growth on malaria transmission due to changes in household preventive behaviours.^36^ Compared to COI analyses, econometric studies are more complex and can incorporate economic adjustment mechanisms. However these studies generally assume that malaria control or particular malaria control interventions are funded by external donors,^16,36^ and thus do not consider the effects that changes in investment levels may have on savings and thus on investments in other production factors. Whereas investing in malaria control contributes to reduce the number of lives lost and work absenteeism because of illness, and thereby increase the size and quality of the labour force, it reduces capital accumulation. At the same time, a decline in morbidity reduces treatment costs and thus mitigates capital accumulation loss. Thus it is not clear how investment in malaria control ultimately affects economic growth through changes in labour force and physical accumulation.

 The current study uses World Health Organization’s (WHO’s) Economic Projections of Illness and Costs macroeconomic model (EPIC) to estimate the impact of malaria control on projected GDP from (*i*) changes in malaria mortality and morbidity on the size of the labor force augmented by educational returns and work experience accumulated over time, (*ii*) changes in the accumulation of physical capital due to reduced savings from increased investments in malaria control, and (*iii*) changes in treatment costs from reduced morbidity. For this EPIC application, the model is adapted to account for the effects of changes in malaria morbidity among children and young adolescents on working adult productivity. As malaria burden reductions and funding levels have stagnated since the launch of the global malaria strategy for 2016-2030, the current study aims at estimating the potential gains in projected GDP that could have been achieved across 26 high burden countries if progress in malaria control and associated investments had matched the vision of the global malaria strategy for 2016-2030.^38^

## Methods

###  Economic Projections of Illness and Costs Modelling Framework

 EPIC was originally developed by the WHO and subsequently adapted and applied to tuberculosis^37,39^ and selected non-communicable chronic diseases.^40-43^ The model quantifies the macroeconomic consequences of investing in health based on a yearly recursive production function accompanied by the evolution of two production factors: the effective labour supply and the physical capital. First, health improvements from intensified disease control increase the stock of the labour force composed of different age groups of workers that have different levels of education and skills accrued over time. Reductions in mortality increase the number of working-age individuals while reductions in morbidity increase their productivity. Productivity losses are measured in terms of years lost to disability (YLDs). One YLD represents one full year of healthy life lost due to ill health and is assumed to be equivalent to one year of full productivity lost. Second, the accumulation of physical capital (tangible assets used in production) depends on the depreciation rate of the stock of physical capital and on savings that is the amount of disposable income saved rather than consumed. Changes in domestic spending due to investments in health interventions (net of the external donor share for these investments) are assumed to be partly financed by savings and thereby reduce the total stock of physical capital. Supplementary file 1 describes the technical specification of the EPIC model.

###  Adaptation of EPIC to Malaria

 While malaria affects all age groups in endemic countries, it disproportionally affects young children mortality and morbidity, typically under the age of five years in countries where transmission is intense (eg, sub-Saharan Africa). Older children (5-9 years old) and young adolescents (10-14 years old) are also at higher risk of malaria than older age groups (15 and above), notably because of immunological and hormonal factors. We adapted EPIC to capture these effects including the effects of caring for children and young adolescents infected by malaria on adult productivity in addition to the effects of malaria infections in working-age adults on labour productivity (Supplementary file 1). We differentiated the effects of malaria morbidity in young children and in older children and young adolescents on adult productivity. In addition, the number of averted deaths in these age groups were subsequently added to the future stock of labour once they reach the age of 15 while considering mortality risks from other diseases. We also developed an analytical approach to estimate the effects of changes in malaria morbidity and mortality on GDP (Supplementary file 1). This EPIC application used R v4.3 and RStudio Cherry Blossom Release (2023.03.1).^44,45^

###  Scope of the Analysis

 To quantify the potential macroeconomic impact of reaching the burden reduction targets set out in the global malaria strategy for 2016-2030,^38^ we compare the projected aggregated GDP of 26 malaria endemic countries over the 2016-2030 period between two scenarios: a business-as-usual scenario in which the coverage of key malaria interventions is sustained at their 2015 level (“Sustain” scenario) and the scale-up scenario in which intervention coverage levels increase between 50% and 90% of the population in needs depending on the intervention considered (“Scale-up” scenario). The two scenarios thus differ in terms of the level of investment required to sustain or scale-up intervention coverage. Considering population growth, the level of investments needed under the Sustain scenario increase at a slower rate than under the Scale-up scenario.

 All malaria control interventions considered in the global strategy for malaria 2016-2030 are considered, including long-lasting insecticidal nets and complementary vector control interventions, seasonal malaria chemoprevention in children, intermittent preventive treatment of pregnant women, diagnostics by blood testing and treatment of confirmed cases, and surveillance activities such as routine epidemiological and entomological information systems. Information on the modelled interventions and coverage scale-up rates is provided inTable S1 (Supplementary file 1). The malaria vaccine recommended by WHO since 2021 and subsequently prequalified in the middle of 2022 is not considered in this analysis.

 Twenty-six countries, which together accounted for more than 90% of the global number of malaria cases and deaths in 2016 were included in the analysis. Of these, two countries including Nigeria and the Democratic Republic of the Congo accounted for nearly 40% of the global malaria burden (Table S2, Supplementary file 1). Of the 26 countries considered, 16 were categorized as low-income countries and 10 as middle-income countries, including nine lower-middle income countries and one higher-middle income country.

###  Data Sources and Analysis

 All data sources are summarized in Table S3, Supplementary file 2.^24,46-54^ Investment need and health impact estimates were obtained from linked modelling work conducted to inform the development of the global malaria strategy 2016-2030. Estimates on the total investment needs per year and per country (country-specific annual total costs) were obtained for each scenario in constant 2014 US$ from Patouillard et al^48^ (Supplementary file 2).We subtracted from these estimates the amount assumed to be financed by external donors, using data on the share of donor funding in total malaria expenditures for each country available from the Global Health Expenditure Database.^24^ We assumed that in each country the share of donor funding stayed constant at 2016-2020 average level throughout the study period. We then assumed that the total cost of the Sustain scenario, net of external donor funding would be funded by domestic consumption while the incremental cost of the Scale-up scenario (net of donor funding) would be paid out by both domestic consumption and savings. In a base-case analysis, 10% of the incremental cost of the Scale-up scenario were assumed to be paid out by savings and 90% by domestic consumption, a situation that is not uncommon in many low- and middle-income countries.^24^ We varied these assumptions in sensitivity analysis (see later).

 Health impact estimates were obtained by combining malaria burden projections from WHO^48^ and from Griffin et al.^50^ Griffin et al developed a malariatransmission model to quantify the potential reductions in malaria burden in the Sustain and Scale-up scenarios, as envisaged by the global malaria strategy 2016-2030 (Supplementary file 2). These modelled mortality estimates were available under each scenario for population groups aged under and above five years of age. We distributed these data proportionally into five-year age groups using WHO’s mortality projections.^48^ In addition, for morbidity estimates, we converted the effects of malaria control on YLD using the ratio of the impact on malaria deaths modelled by Griffin et al to those projected by the WHO.^48^ To model morbidity and mortality effects on the effective labour supply, we then merged five-year age groups from the age of 15 and above into age groupings used in ILO labor force participation rate dataset (15-29, 30-44, 45-59, 60-64, 65-69)^47^ (Supplementary file 2).

 To transfer malaria morbidity in children and young adolescents on the productivity of working adults, we assumed that one year lost to child morbidity in the 0-4 age group and in the 5-14 age group was equivalent to one full year and 0.5 year productivity loss in working adults, respectively.^16,17,35,51,52,55-57^ We varied these assumed transfer rates in sensitivity analysis (see below).

 EPIC’s parameters including the saving rate (percentage of disposable income saved rather than spent on consumption), the growth rate of total factor productivity (the change in economic growth that occurs due to factors other than changes in the labour force and capital stock), the output elasticity of physical capital (the change in the output that results from a change in physical capital), the growth rate of educational capital (returns to education that increase the quality of the labour force) and the depreciation rate (the percentage decrease in the monetary value of tangible assets over time) for each country were derived from the Penn World Table 10.01 (Supplementary file 2).^46^ Missing values for country-specific data extracted from published sources were imputed using the mean of data in countries from the same income group.

 For each country, macroeconomic parameters were assigned a normal distribution informed by their respective mean and standard deviation over the 2005-2014 period (Table S4 and Figure S1, Supplementary file 2) and were combined with investment need and health impact estimates to generate 1000 estimated projections of annual GDP for each scenario and country over the 2016-2030 period.

 We calculated the mean difference and 95% uncertainty intervals (UIs) in annual GDP between the two scenarios for each country. We summarized results as percentage differences in GDP aggregated across the 26 countries and according to World Bank country income groups, per year and for the entire study period. We calculated the relative contribution of morbidity and mortality changes to the difference in GDP between the two scenarios (Supplementary file 1).

 These main results are presented for a base-case analysis in which 10% of incremental investment needs would be paid out of savings in the Scale-up scenario while in both scenarios, the morbidity transfer rate of one YLD in the 0-4 age group would be equivalent to one full year productivity loss in working adults and in the 5-14 age group to 0.5-year productivity loss in working adults.

###  Sensitivity Analysis

 Given the published estimates on the effect of malaria on labour productivity across different settings, age groups and occupations, we varied the morbidity transfer rates from 1 to 0.6 for one year lost to morbidity in the 0-4 age group and from 0.5 to 0.3 for one year lost to morbidity in the 5-14 age group. The percentage of investment needs (net of external donor funding) paid out by domestic savings in the Scale-up scenario was varied from 10% to 50% and from 10% to 90%. These increases in the proportion of incremental domestic investments needs paid out by savings may correspond to situations in which governments cannot raise sufficient revenues, out of taxation from example, such that a larger share of investment needs is paid out from savings. Thus, it was assumed that in the Scale-up scenario, intensifying malaria control does not rely on increasing household consumption given the significant share of malaria expenditures that are already paid out of pocket by households in many low- and middle-income countries.^24^

## Results

 Across all 26 countries and over the entire study period, an additional 12 million malaria related deaths and 60 million YLD to malaria could be averted under the Scale-up scenario compared to the Sustain. Forty percent of the averted burden would take place in three of the 26 countries (Nigeria, India, and the Democratic Republic of Congo), reflecting their large population size and malaria burden in absolute terms. Net of assumed external donor funding, total domestic investment needs were estimated at US$ 22.92 billion under the Sustain scenario (64% of total scenario investment needs) and US$ 45.17 under the Scale-up scenario (61% of total scenario investment needs) (Figure S2, Supplementary file 3). Under both scenarios, nearly 70% of total domestic investment needs were estimated in four of the 26 countries studied: India (about 30%), Nigeria (about 25%) and the Democratic Republic of Congo and the United Republic of Tanzania (each about 6%).

###  Estimated Total Macroeconomic Impact 

 In the base-case analysis, the macroeconomic dividend from scaling-up malaria control as set out by the global malaria strategy 2016-2030 was estimated at US$ 152.50 billion (95% UI 152.00-153.00) in total across all 26 modelled countries over the study period.

 These estimated gains would be equivalent to a 0.1750% increase in total GDP projected for the study period for all 26 countries (Table S5), with around 95% of the mean total GDP gain attributable to averted malaria morbidity (Table S6). Across the 16 low-income countries, the economic dividend was estimated to be higher, equivalent to 0.3193% in total GDP projected for the study period while in the nine lower-middle income countries, it would be about half at 0.1567% of total projected GDP. Gains were estimated to be higher in Sub-Saharan countries, at 0.5684% of projected GDP in Niger, 0.4315% in Mozambique, 0.4144% in Mali and 0.3541% in Nigeria, for example. Outside sub-Saharan African countries where the burden of malaria on children and young adolescent is relatively smaller, gains would be lower, such as, for example, in India at 0.1149% of projected GDP.

 Assuming lower transfer rates of children morbidity on working adult productivity across all 26 modelled countries, the mean total gain over the study period declined slightly but was still significant, equivalent to 0.1439% of total GDP projected over the study period (Table S7). In the above-mentioned sub-Saharan African countries, gains would be 0.05 to 0.20% point lower than in the base-case analysis, at 0.3812% of projected GDP in Niger, 0.3542% in Mozambique, 0.2913% in Mali and 0.2714% in Nigeria, for example. By contrast, in India, the gain would not change very much compared to the base-case analysis (0.1014% of projected GDP), reflecting the relatively lower burden of malaria in children and younger adolescents in this country and thus a lower sensitivity to changes in the morbidity transfer rates.

 Finally, increasing the percentage share of domestic investments paid out from savings from 10% to 50% and from 10% to 90% under the Scale-up scenario reduced the economic dividend from 0.1750% to respectively 0.1391% and 0.1031% of total GDP projected over the study period (Tables S8 and S9).

###  Estimated Macroeconomic Impact Over Time

**Figure 1:**
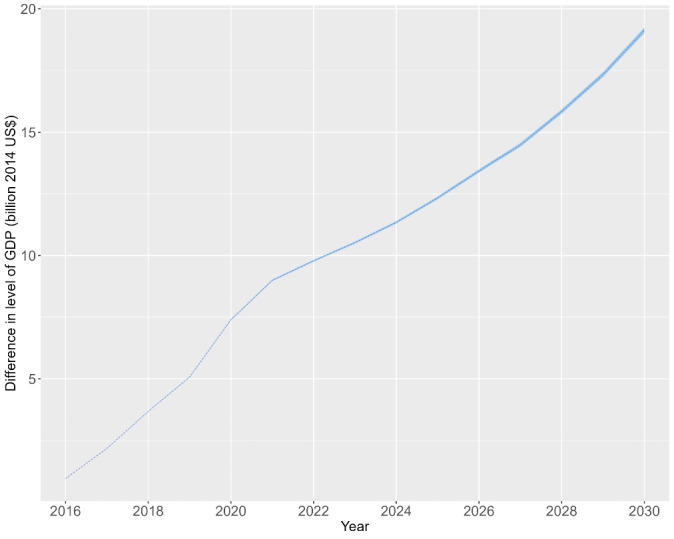
Annual Gains in Projected GDP (2014 Billion US$ and 95% UI) for All 26 Modelled Countries Between 2016 and 2030. Trend in annual gains in GDP projected for all modelled countries between 2016 and 2010 in the Scale-up scenario compared to the Sustain scenario. Thicker blue line shows greater uncertainty using 95% UIs. Abbreviations: UI, uncertainty interval; GDP, gross domestic product.

**Figure 2:**
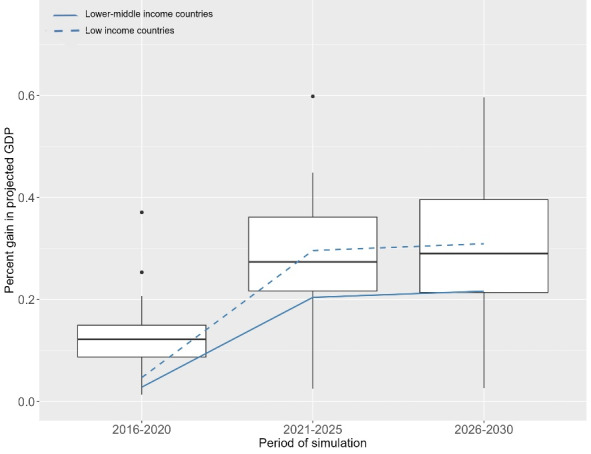
Percent Gain in Projected GDP in the Scale-up Scenario Compared to the Sustain Scenario, Across All 26 Countries in 2016-2020, 2021-2025 and 2026-2030 and Trend in Projected GDP Gain Across 16 Low-Income Countries and 9 Low-Middle Income Countries Between 2016 and 2030 (Base-Case Analysis).Boxplots represent the distribution of percent gains (median and upper and lower quartiles) in projected GDP across 26 individual modelled countries for each 5-year period. Whiskers indicate the variability in results outside upper and lower quartiles. Dots indicate individual point estimate outliers. Dashed and solid lines indicate the arithmetic mean of the gain in projected GDP for 16 low-income countries and the 9 low-middle-income countries respectively. Abbreviation: GDP, gross domestic product.

 Across all 26 countries, macroeconomic annual benefits would increase over time (Figure 1). The mean gains would be equivalent to 0.0858% in total projected GDP between 2016 and 2020, 0.1855% between 2021 and 2025 and 0.2152% between 2026 and 2030. In the 16 low-income countries, mean gains were estimated at 0.1380% of projected total GDP between 2016 and 2020, 0.3278% between 2021 and 2025 and 0.3798% between 2026 and 2030 and in the 9 lower-middle income countries at 0.08%, 0.17% and 0.19% over the three periods respectively (Figure 2). Trends in projected GDP annual gains (in billion US$ and percentages) over the entire study period by country income group are available in Figures S3 and S4.The relative contribution of averted mortality to the gain in projected GDP increased over the study period, reflecting the increasing number of children reaching working-age (Figure S5), with additional gains expected beyond the study period.

## Discussion

 We used the WHO’s EPIC macroeconomic model to estimate the potential gain in projected GDP of reducing the burden of malaria in 26 high malaria burden, as set out by the global malaria strategy for 2026-2030. For this application of EPIC to malaria, a disease responsible for significant mortality in young children and ill-health throughout life, we adapted the model to account for, not only the direct effects of childhood mortality and adult morbidity on the effective labour supply but also the effects of morbidity in childhood and early adolescence on the productivity of working adults. In our base-case analysis, scaling-up malaria control to reach the global burden reduction targets for 2016-2030 could have generated economic gains of around 0.17% in projected GDP across all 26 countries, with these potential gains increasing over the time period. These gains were estimated to be, on average, nearly twice higher (0.32%) in low-income countries, including countries with some of the highest malaria burden in the world. Potential gains in projected GDP over the studied period were found to be sensitive to changes in key assumptions. First, assuming lower morbidity transfer rates from children and young adolescents to working-age adults reduced the estimated gains, which were still significant at 0.14% of projected GDP between 2016 and 2030 across all 26 countries. Second, greater reliance on domestic savings for malaria investments decreased potential gains down to 0.14% or 0.10% of projected GDP across all modelled countries, depending on the assumptions made. Despite these sensitivities to changes in key assumptions, gains in the low-income country groups and in countries with the highest burden of malaria remained above the average gain estimated in the base-case analysis. These results imply that, under the modelled assumptions, the health benefits of reducing malaria could outweigh the economy-wide opportunity cost of malaria control, in terms of the productive potential of these investments in other important areas.

 Given the stagnating levels in malaria investments and burden since 2015, it is evidently unlikely that the economic gains estimated in this study would materialize by 2030, including if malaria control efforts were to intensify dramatically over the next decade. Our analysis did not consider the disruptions of the COVID-19 pandemic on malaria control, which resulted in an increased number of malaria cases in 2020 and 2021. Thus, whilst global trends in malaria case incidence and deaths have been broadly stable since 2015, notably due to a slight decline in the burden of malaria in 2018, the estimated potential GDP gains should be seen as retrospective estimates of the direction and potential magnitude of the economic benefits associated with the global malaria strategy for 2016-2030. Focusing on 26 countries instead of the 84 countries with malaria endemicity in 2021 allowed us to focus on the potential economic benefits of malaria control in the highest burden and poorer endemic countries. In low malaria burden or elimination settings, the interruption of malaria transmission requires expanding the range of interventions which often imply high costs, greater uncertainty, and risk of failure whilst the incremental health benefits decline, bringing additional challenges in the interpretation of results from benefit-cost analyses.^25,58^

 Various methodological approaches have been used to estimate the economic value of malaria control. While these approaches have their own strengths and limitations, EPIC offers an alternative analytical framework to conduct investment cases in health that can account for the opportunity costs of health interventions from an economy-wide perspective. The usefulness and flexibility of the EPIC framework have been demonstrated in earlier applications to selected non-communicable diseases and to tuberculosis.^37,39-43^ For example, a recent EPIC application estimated that the worldwide economic burden of cancers could represent an annual tax on global GDP of 0.55% between 2020 and 2050.^42^ Another recent EPIC-based study estimated that introducing a novel tuberculosis vaccine for infants and for adolescents and adults could generate an economic dividend equivalent to an increase in projected GDP of, respectively, 0.004% and 0.033% across 105 low- and middle-income countries between 2028 and 2080.^37^ Focusing on the recent malaria literature, a macroeconomic modelling study accounting for changes in household preventive behaviour estimated that malaria vaccination in children below five years of age could increase Ghana’s GDP by 0.5% per year over a 30-year period assuming 100% vaccine coverage and external funding.^16^ Also in Ghana, a similar model was used to explore the potential for existing malaria control interventions alongside economic development to achieve malaria elimination.^36^ Given the differences in methods, it is challenging to make comparative observations on results. ^36^ In terms of methodological approach, EPIC offers a relatively simple analytical framework that can be easily adapted to different diseases and interventions in one or several countries. Whilst our application did not integrate demographic, epidemiologic, costing and macroeconomic models in a shared framework, it used health impacts and resources needs estimates stemming from linked epidemiologic and costing models, while accounting for the opportunity costs of malaria control interventions, one aspect not considered to date in other macroeconomic models applied to malaria and of relevance for priority setting. Future work will aim to integrate EPIC to the suite of methodologies that WHO develops to support value for money assessments and address the common resource and capacity gaps for conducting economic evaluations in many countries.^59-61^

 Our study has some limitations. Whilst by using a standard augmented Solow framework we conform to well-accepted norms in economic modelling, EPIC does not account for endogenous changes that may occur on key parameters due to changes in health status. The population growth rate, saving behaviour and thus the accumulation of physical capital, as well as human capital accumulation and future labour productivity may be affected by changes in health interventions and associated improvements in health status over time. While our study period is relatively short, which likely mitigates the impact of such changes during the study period, future applications of EPIC could consider modelling household behaviours and decision-making over time. In addition, the specification of the manner in which health investments affect capital accumulation could draw on an improved national accounting identity framework.

## Conclusion

 Our results offer insights on the benefits that investing in malaria control can have beyond health. More generally, it shows that the EPIC modelling framework offers a simple approach that may be adapted to different diseases and interventions for investment cases in health.

## Acknowledgment

 We thank the members of the WHO’s Scientific Advisory Group on Malaria Eradication for insightful advice.

## Ethical issues

 No ethical approval was sought as this is secondary data analysis.

## Competing interests

 Edith Patouillard is staff member of the WHO. Other authors declare that they have no competing interests.

## Disclaimers

 The views expressed are those of the authors and do not necessarily represent the views of their respective organizations.

## Funding

 Funders include the WHO and the Bill & Melinda Gates Foundation. The funders had no role in the design, writing or the decision to submit the manuscript for publication.

## Supplementary files

Supplementary file 1. EPIC Technical Specification and Adaptation to Malaria.

Supplementary file 2. Data Sources.

Supplementary file 3. Results.
