# Supplementary material for: The Macroeconomic Impact of Increasing Investments in Malaria Control in 26 High Malaria Burden Countries: An Application of the Updated EPIC Model
Source: Int J Health Policy Manag. 2023 Oct 4;12:7132. doi: 10.34172/ijhpm.2023.7132 (PMC10590221; doi:10.34172/ijhpm.2023.7132)
Supplement: Supplementary file 1 — EPIC Technical Specification and Adaptation to Malaria. [file ijhpm-12-7132-s001.pdf]

Article title: The Macroeconomic Impact of Increasing Investments in Malaria Control in 26 High Malaria Burden Countries: An Application of the Updated EPIC Model

Journal name: International Journal of Health Policy and Management (IJHPM)

Authors' information: Edith Patouillard[1]*, Seoni Han[2], Jeremy Lauer[3], Mara Barschkett[4], Jean-Louis Arcand[5,6,7,8]

[1]Department of Health Financing and Economics, World Health Organization, Geneva, Switzerland.

[2]Korea Institute for International Economic Policy, Sejong, Korea.

[3]Strathclyde Business School, University of Strathclyde, Glasgow, UK.

[4]Federal Institute for Population Research and Department of Public Economics, German Institute of Economic Research (DIW Berlin), Berlin, Germany.

[5]Department of International Economics, The Graduate Institute, Geneva, Switzerland.

[6]Global Development Network, New Delhi, India.

[7]Mohammed VI Polytechnic University, Rabat, Morocco.

[8]Foundation for Studies and Research on International Development (FERDI), Clermont Ferrand, France.

***Correspondence to**: Edith Patouillard, Email: patouillarde@who.int

Citation: Patouillard E, Han S, Lauer J, Barschkett M, Arcand JL. The macroeconomic impact of increasing investments in malaria control in 26 high malaria burden countries: an application of the updated EPIC model. Int J Health Policy Manag. 2023;12:7132. doi:10.34172/ijhpm.2023.7132

Supplementary file 1. EPIC Technical Specification and Adaptation to Malaria

1. Technical specifications of the EPIC macroeconomic model 2
2. EPIC technical adaptation for malaria control application 4
3. Relative contribution of malaria mortality and morbidity reductions to total economic output 5
4. Scope of the study 6

Table S1: Modelled coverage levels, by intervention, under "Sustain" and "Scale-up" scenarios 6

Table S2: Name of studied countries and percentage of global malaria cases and deaths in each country in 2016 7

1. Technical specifications of the EPIC macroeconomic model

The WHO EPIC (Economic Projections of Illness and Cost) model employs a human capital augmented Solow model with a linear-homogenous Cobb-Douglas production function:

$$Y_{it} = \gamma_i A_{it} * K_{it}^{\alpha_{it}} * (H_{it} * L_{it})^{1-\alpha_{it}} \text{ where } 0 < \alpha < 1, \quad (1)$$

where Y_{it} is the GDP of country i in year t, K is the physical capital, A_{it} is Total Factor Productivity (TFP), L_{it} is the labor force, H_{it} is an educational capital index, α_{it} is the elasticity of output with respect to physical capital and γ_i a scaling factor.

A_{it} refers to technological advancements and other factors such as resource endowment, climate, institutions, etc. that contribute to the GDP in addition to contributions from the labor force and the physical capital. TFP is here Hicks-neutral and enters multiplicatively in the production function as an increasing scale factor. The scaling factor γ_i is a calibration parameter used to fit the model to reported GDP values so that reported and estimated values are identical in the first year of the projection.

The product $H_{it} * L_{it}$ represents the stock of labor augmented by education. H_{it} is a country-specific educational capital index that accounts for the returns to education that augment the quality of the labor force.

α_{it} is the change in output resulting from a change in physical capital and is denoted by $\alpha_{it} \in (0, 1)$. The elasticity of output with respect to labor is thus denoted by $(1 - \alpha_{it}) \in (0, 1)$.

The contributions of the physical capital and of the labor force are scaled with the level of technology in each country. Each country has different initial level of technology and the TFP is assumed to grow at rate g_{it}

$$A_{it} = A_{i(t-1)} e^{g_{it}} \quad (2)$$

Physical capital accumulation

The law of motion for physical capital is given by the equation:

$$K_{it} = s_{it} Y_{i(t-1)} + (1 - \delta_{it}) K_{i(t-1)} \quad (3)$$

where s_{it} is the savings rate of country i in year t and δ_{it} the depreciation rate of country i in year t. s_{it} and δ_{it} are here exogenously given.

The saving rate s_{it} is the percentage of disposable income saved rather than spent on consumption and is calculated using the real GDP and consumption data for country i at time t. The depreciation rate δ_{it} is the percentage reduction in the monetary value of assets over time.

The initial level of the stock of physical capital per efficiency unit of labor is calibrated to correspond to its steady-state level k_{it}^* given by:

$$k_{it}^{*} = (\frac{s_{it}\gamma_i A_{it}}{n_{it} + h_{it} + \delta_{it}})^{\frac{1}{1-\alpha_{it}}}, \quad (4)$$

where $k \equiv K/HL$. This avoids unrealistically high growth rates generated by what would correspond to transitional dynamics toward the steady state at the start of the simulation, and therefore yields the balanced growth path.

Labor force

The labour force is defined as the number of individuals between 15 and 69 years of age who participate in the labour market. The raw size of the labor force in each age group was calculated according to:

$$L_{ait} = p_{ai} N_{ait}. \quad (5)$$

where L_{ait} is the stock of the labor force in each age group, p_{ai} is the labor force participation rate for age group a (15-29, 30-44, 45-59, 60-64), and N_{ait} is the base population for the relevant age group.

These age-group specific stocks of labor were then augmented by experience and skill according to:

$$L_{it} = \sum_{a=15}^{60} \rho_a L_{ait}, \quad (6)$$

Where L_{it} is the skill -augmented stock of the aggregate labor force, L_{ait} is the stock of labor force in each age group and ρ_a is the average skill-augmenting factor for age group a that adjusts for work experience over time for different age group according to:

$$\rho_a = \rho_A + \rho_B(age_a - 15) + \rho_C(age_a - 15)^2 \quad (7)$$

where age_a is the age of a worker, and ρ_A = 0.8, ρ_B = 0.02, and ρ_C = -0.0002 (see reference).

In summary, the size of the labor force is augmented by education and, over time, work experience according to:

$$H_i L_{it} = H_i \sum_{\alpha=15}^{60} \rho_a L_{ait} \quad (8)$$

Consequences of investing in disease control interventions

Investing in disease control interventions is assumed to affect the evolution of the physical capital. **Eq (3)** can be rewritten as:

$$K_{it} = s_{it} Y_{i(t-1)} - rC_{it} + (1 - \delta_{it}) K_{i(t-1)}, \quad (9)$$

where C_{it} is the amount invested and paid out from domestic funding sources, net of treatment cost savings stemming investments in disease control interventions and *r* is the proportion of investments *C* paid out from domestic savings. The remainder of the investments is assumed to be paid out from domestic consumption.

Health status improvements that result from disease control interventions is assumed to affect the effective labor force. **Eq (6)** can be rewritten as:

$$L_{it} = \sum_{a=15}^{60} \rho_a \; L_{ait} + z_{it} + b_{it} \quad (10)$$

where z_{it} is the number of deaths averted and b_{it} is the number of Years Lost to Disability (YLDs) averted at time t in country i.

Productivity losses due to illness are measured in terms of Years Lost to Disability (YLDs). One YLD is assumed to be equivalent to one year of full productivity lost. Averted productivity losses (productivity gains) are measured as the difference in YLD between two scenarios.

Reference:

Cuddington JT, Hancock JD. Assessing the impact of AIDS on the growth path of the Malawian economy. J Dev Econ. 1994;43(2):363-8.

2. EPIC technical adaptation for malaria control application

The EPIC model was adapted to capture the spillover effects that reduced malaria in childhood and young adolescence may have on the effective labor force.

Consequences of malaria control

EPIC was adapted to account for the effects that improved children and young adolescent's health may have on the size and quality of the labor force. First, children and young adolescents aged 0 to 14 years old, who are affected by malaria, were assumed to adversely affect the productivity of adults. Conversely, reductions in morbidity in this age group were assumed to translate in productivity gains (or averted productivity losses) in the working adult population b_{it}, according to:

$$b_{it} = \sum_{a=15}^{60} \rho_a b_{ait} + \rho_{30}(q_1 b_{0it} + q_2 b_{5it}) \quad (11)$$

where b_{ait} is the number of averted YLD among working adults of age group a at time t in country i , ρ_a is the skill-augmenting factor that incorporates the effects of averted YLD among working adults in age group a on their average work experience , $q_1 b_{0it} + q_2 b_{5it}$ b_{0it} and b_{5it} are the numbers of averted YLD among children and young adolescents aged 0-4 and 5-14 year old respectively that affect the productivity of working adults, $q_1$and q_2 are the "transfer rates" defined as the share of averted YLD in children and adolescents aged 0-4 and 5-14 respectively that are transferred to adults as productivity gains.

Second, the number of averted deaths in 0- to 14-year-old children and adolescents were assumed to be added to the future stock of labour when children and adolescents reach the age of 15, according to:

$$z_{it} = \sum_{a=15}^{60} \rho_a z_{ait} + \rho_{15}\left(z_{0i,t-13} + z_{5i,t-5}\right) \quad (12)$$

The first term $\rho_a z_{ait}$ is the number of deaths averted in working adults in age group a multiplied by ρ_a, the experience augmenting factor for age group a. The second term $\rho_{15}\left(z_{0i,t-13} + z_{5i,t-5}\right)$ represents

the gain in the size of the labour force stemming from the number of averted deaths in young age groups once they reach 15 years of age augmented by experience. The gain in labour force size considers mortality risks from other diseases using all-cause mortality data excluding malaria. The number of averted deaths in children aged 0 to 4 years at year $t - 13$ is added to the labour stock at year t, with , $t - 13$ reflecting the average number of years for that 0-4 age group to reach age 15. Similarly, the number of deaths averted in children and young adolescents aged 5 to14 year old at year $t - 5$ is added to the stock of labour at year t with $t - 5$ reflecting the average number of years for that age group to reach age 15.

3. Relative contribution of malaria mortality and morbidity reductions to total economic output

For this current EPIC application, we calculated the relative contribution of averted morbidity and mortality to total economic gain. As we cannot separate investments that contribute to mortality to investments that contribute to morbidity, we first estimated the total economic gain from malaria burden reductions excluding the cost of interventions under each scenario and calculated the share of the total gain (excluding intervention cost) that could be attributed to mortality and morbidity reduction respectively. We then used these shares of economic gains attributable to mortality and morbidity respectively and multiplied them by the total economic gain accounting for the effects of intervention costs.

4. Scope of the study

Table S1: Modelled coverage levels, by intervention, under "Sustain" and "Scale-up" scenarios
In both scenarios, routine epidemiological and entomological information systems were also included

Scenario	Intervention	Baseline	2020 coverage targets	2025 coverage targets	2030 coverage targets
**"Sustain"**	All	Maintain coverage of all existing interventions at their 2015 level			
**"Scale-up"**	Vector control, incl LLIN and IRS	country-specific coverage	80% with LLIN replaced every 3 years	90% with LLIN replaced every 2 years	Maintain coverage
		10% additional coverage with complementary control measures for resistance management			
	SMC	0%	80%	95%	Maintain
	IPTp	0%	80%	90%	Maintain
	Blood tests, using RDTs or microscopy	20%			90%
		10% G6PD testing			90%
	Treatment of uncomplicated cases	2013 country-specific coverage	90% at public facilities, 50% in communities		75% community-based treatment
	Treatment of severe cases	100% hospitalized cases treated with quinine	100% hospitalized cases treated with injectable artesunate	50% severe cases with rectal artesunate in communities	75% severe cases with rectal artesunate in communities

LLIN: Long-Lasting Insecticide Bednets; IRS: Indoor Residual Spraying; SMC: Seasonal Chemoprevention in Children;
IPTp: Intermittent Preventive Treatment in pregnant women; RDT: Rapid Diagnostic Test

References:

49.Patouillard E, Griffin J, Bhatt S, Ghani A, Cibulskis R. Global investment targets for malaria control and elimination 2016-2030. *BMJ Global Health*. (2 (2) e000176)doi:DOI: 10.1136/bmjgh-2016-000176

50.Griffin JT, Bhatt S, Sinka ME, et al. Potential for reduction of burden and local elimination of malaria by reducing Plasmodium falciparum malaria transmission: a mathematical modelling study. *Lancet Infect Dis*. Apr 2016;16(4):465-72. doi:10.1016/S1473-3099(15)00423-5

Table S2: Name of studied countries and percentage of global malaria cases and deaths in each country in 2016

Country name	% of global malaria cases	% of global malaria deaths
Nigeria	27%	23%
Democratic Republic of the Congo	10%	14%
India	6%	5%
Mozambique	4%	3%
Ghana	4%	3%
Mali	4%	5%
Burkina Faso	4%	5%
Niger	4%	4%
Uganda	4%	3%
United Republic of Tanzania	3%	4%
Cameroon	3%	2%
Côte d'Ivoire	2%	2%
Guinea	2%	2%
Rwanda	2%	1%
Malawi	2%	2%
Kenya	2%	2%
Angola	2%	3%
Benin	1%	1%
Togo	1%	1%
Sierra Leone	1%	2%
Chad	1%	2%
South Sudan	1%	1%
Burundi	1%	1%
Central African Republic	1%	1%
Papua New Guinea	1%	1%
Pakistan	1%	0%
All	93%	93%

Reference:

WHO. *World Malaria Report 2017.* Geneva: World Health Organization; 2017.
