# Supplementary material for: The Macroeconomic Impact of Increasing Investments in Malaria Control in 26 High Malaria Burden Countries: An Application of the Updated EPIC Model
Source: Int J Health Policy Manag. 2023 Oct 4;12:7132. doi: 10.34172/ijhpm.2023.7132 (PMC10590221; doi:10.34172/ijhpm.2023.7132)
Supplement: Supplementary file 2 — Data Sources. [file ijhpm-12-7132-s002.pdf]

Article title: The Macroeconomic Impact of Increasing Investments in Malaria Control in 26 High Malaria Burden Countries: An Application of the Updated EPIC Model

Journal name: International Journal of Health Policy and Management (IJHPM)

Authors' information: Edith Patouillard[1]*, Seoni Han[2], Jeremy Lauer[3], Mara Barschkett[4], Jean-Louis Arcand[5,6,7,8]

[1]Department of Health Financing and Economics, World Health Organization, Geneva, Switzerland.

[2]Korea Institute for International Economic Policy, Sejong, Korea.

[3]Strathclyde Business School, University of Strathclyde, Glasgow, UK.

[4]Federal Institute for Population Research and Department of Public Economics, German Institute of Economic Research (DIW Berlin), Berlin, Germany.

[5]Department of International Economics, The Graduate Institute, Geneva, Switzerland.

[6]Global Development Network, New Delhi, India.

[7]Mohammed VI Polytechnic University, Rabat, Morocco.

[8]Foundation for Studies and Research on International Development (FERDI), Clermont Ferrand, France.

***Correspondence to**: Edith Patouillard, Email: patouillarde@who.int

Citation: Patouillard E, Han S, Lauer J, Barschkett M, Arcand JL. The macroeconomic impact of increasing investments in malaria control in 26 high malaria burden countries: an application of the updated EPIC model. Int J Health Policy Manag. 2023;12:7132. doi:10.34172/ijhpm.2023.7132

Supplementary file 2. Data Sources

1. EPIC parameters and data sources 2

Table S3: EPIC parameter and corresponding variable name and description with data sources........ 2

2. Trends in macroeconomic parameters between 2005 and 2014 4

Figure S1: Trends in four macroeconomic parameters between 2005 and 2014, by country 5

Table S4: Variance-covariance matrix of the four macroeconomic parameters 5

1. EPIC parameters and data sources

Table S3: EPIC parameter and corresponding variable name and description with data sources.

Parameter	Variable name and description	Data sources and/or References
Y_{it}	Gross domestic product (GDP), indicator *NY.GDP.MKTP.KD,* 2015 initial value	54
K_{it}	Capital stock, variable name in data source: rnna. Initial stock of capital assumed to b the stock in year 2014	46
α_{it}	Elasticity of output with respect to physical capital, variable name in data source: labsh	46
s_{it}	Saving rate estimated from real GDP, variable rgdpna and real consumption, variable name in data source: rconna	46
δ_{it}	Average depreciation rate of the capital stock, variable name in data source: delta	46
h_i	Human capital index, based on years of schooling and returns to education, variable name is data source: hc	46
A_{it}	Total Factor Productivity, variable name in data source: rtfpna	46
N_{ait}	Population data divided into eight age groups: 0-4, 5-14, 15-29,30-44, 45-59, 60-69, 70-79, 80 and above	54,48
p_{ai}	Labor force participation rate for four age groups: 15-29, 30-44, 45-59, 60-64, 65-69, variable name in data source EAP_2WAP_SEX_AGE_RT_A	47
C_{it}	Estimated amount of investments in disease control interventions paid out by domestic sources, net of treatment cost savings stemming from disease control interventions and net of external donor funding	Authors calculations based on 24 and 49
R	Proportion of investments *C* paid out from domestic savings	Authors assumptions
z_{it}	Number of deaths averted from changes in disease control interventions.	Authors calculations based on 48 and 50
b_{it}	Number of Years Lost to Disability (YLDs) averted from changes in disease control interventions	Authors calculations based on 48 and 50
q_1, q_2	Proportion of YLD lost to malaria in 0-4 age group and 5-14 age group respectively, which are transferred as productivity losses in working adults	Authors calculations based on 16,17,35,51,52,55-27

Data for C_{it} come from a study by Patouillard et al (49) and WHO's GHED (24). Patouillard et al. sourced commodity procurement prices from international databases or from expert consultations for commodities with no prices available. They obtained cost data on freight and insurance, in-country delivery and surveillance from the published literature, available national malaria strategic plans and from National

Malaria Control Programmes reports to the WHO. Patient delivery cost estimates for treatment at health facilities were sourced from the WHO-CHOICE project. Commodity prices were multiplied by the quantities of resources needed to sustain or scale-up malaria control interventions under each scenario. For preventive interventions, quantities were derived from country-specific population in need estimates and targeted coverage levels, accounting for wastage. For curative interventions, quantities in each scenario were derived from the number of malaria cases modelled by Griffin et al. (50) and assumed to receive appropriate care at public health facilities or in the community. Treatment costs associated with false-positive were also included using the reported specificity rate for rapid diagnostic tests. Data from the GHED were used to calculate the amount of investment needs net of donor funding, as described in the main manuscript.

Data on malaria mortality and morbidity were obtained from Griffin et al. (50) and WHO (48). Griffin et al. developed a dynamic epidemiological model of the transmission of malaria to quantify the impacts of the Sustain and Scale-up scenarios on malaria mortality and morbidity by country and year over the 2016-2030 period. Their model considered country specific variations in baseline malaria endemicity, seasonality in transmission, vector species, 2015 intervention coverage levels and population growth. Estimates of malaria morbidity and mortality reductions were combined with WHO data as described in the main manuscript.

References:

16. Yerushalmi E, Hunt P, Hoorens S, Sauboin C, Smith R. Exploring the Use of a General Equilibrium Method to Assess the Value of a Malaria Vaccine: An Application to Ghana. *MDM Policy & Practice*. 2019;

17. Cutler D, Fung W, Kremer M, Singhal M, Vogl T. Early-life Malaria Exposure and Adult Outcomes: Evidence from Malaria Eradication in India. *American Economic Journal: Applied Economics*. 2010;2(2):72-94.

24. WHO. Global Health Expenditures Database. Global Health Expenditure Database Global Health Expenditure Database (who.int)

35. Bleakley H. Malaria Eradication in the Americas: A Retrospective Analysis of Childhood Exposure. *American Economic Journal: Applied Economics*. 2010;2(2):1-45.

46. Feenstra RC, Inklaar R, Timmer MP. The Next Generation of the Penn World Table. *American Economic Review*. 2015;105(10):3150-3182. Data available at PWT 10.01 | Penn World Table | Groningen Growth and Development Centre | University of Groningen (rug.nl)

47. ILOSTAT. Statistics on the population and labour force. Geneva: International Labour Organization; 2015 The leading source of labour statistics - ILOSTAT

48. World Health Organization (WHO). Projection analysis based on Global Health Estimates (GHE) 2016: Deaths by Cause, Age, Sex, by Country and by Region, 2000 – 2016. WHO; 2018

49. Patouillard E, Griffin J, Bhatt S, Ghani A, Cibulskis R. Global investment targets for malaria control and elimination 2016-2030. *BMJ Global Health*. 2016;(2 (2) e000176)doi:DOI: 10.1136/bmjgh-2016-000176

50. Griffin JT, Bhatt S, Sinka ME, et al. Potential for reduction of burden and local elimination of malaria by reducing Plasmodium falciparum malaria transmission: a mathematical modelling study. *Lancet Infect Dis*. Apr 2016;16(4):465-72. doi:10.1016/S1473-3099(15)00423-5
51. Sauerborn R, Shepard DS, Ettling MB, Brinkmann U, Nougtara A, Diesfeld HJ. Estimating the direct and indirect economic costs of malaria in a rural district of Burkina Faso. *Trop Med Parasitol*. Sep 1991;42(3):219-23.
52. Guiguemde TR, Coulibaly N, Coulibaly SO, Ouedraogo JB, Gbary AR. [An outline of a method for estimating the calculated economic cost of malaria cases: its application to a rural area in Burkina Faso (Western Africa)]. *Trop Med Int Health*. Jul 1997;2(7):646-53. Esquisse d'une methode d'estimation du cout economique chiffre des acces palustres: application a une zone rurale au Burkina Faso (Afrique de l'Ouest). doi:10.1046/j.1365-3156.1997.d01-351.x
53. The World Bank. DataBank, World Development Indicators. World Development Indicators | DataBank (worldbank.org)
54. UN Population Division. World Population Prospects. New York: United Nations; 2015. World Population Prospects - Population Division - United Nations
55 Chima RI, Goodman CA, Mills A. The economic impact of malaria in Africa: a critical review of the evidence. *Health Policy*. Jan 2003;63(1):17-36. doi:Pii S0168-8510(02)00036-2 Doi 10.1016/S0168-8510(02)00036-2
56. Asenso-Okyere WK, Dzator JA. Household cost of seeking malaria care. A retrospective study of two districts in Ghana. *Soc Sci Med*. Sep 1997;45(5):659-67. doi:10.1016/s0277-9536(96)00383-8
57 Sauerborn R, Nougtara A, Hien M, Diesfeld HJ. Seasonal variations of household costs of illness in Burkina Faso. *Soc Sci Med*. Aug 1996;43(3):281-90. doi:10.1016/0277-9536(95)00374-6

2. Trends in macroeconomic parameters between 2005 and 2014

The four panels of Figure S1 report the evolution over time of our key macroeconomic parameters (the depreciation rate δ, the investment share s, the capital share α and the growth rate of total factor productivity (TFP), g) for the 26 modelled countries. Blue thick lines are non-parametric smooths. For most countries, the macroeconomic parameters display relatively little variation, although there are some exceptions such as the capital share for Niger, the investment rate for Nigeria, or the growth rate of TFP for several countries. Table S4 displays the covariances between the four macroeconomic parameters. Covariances are all extremely small, as shown by the variance-covariance matrix. As such, our simulation results are essentially unchanged when off-diagonal elements are taken into account.

Figure S1: Trends in four macroeconomic parameters between 2005 and 2014, by country

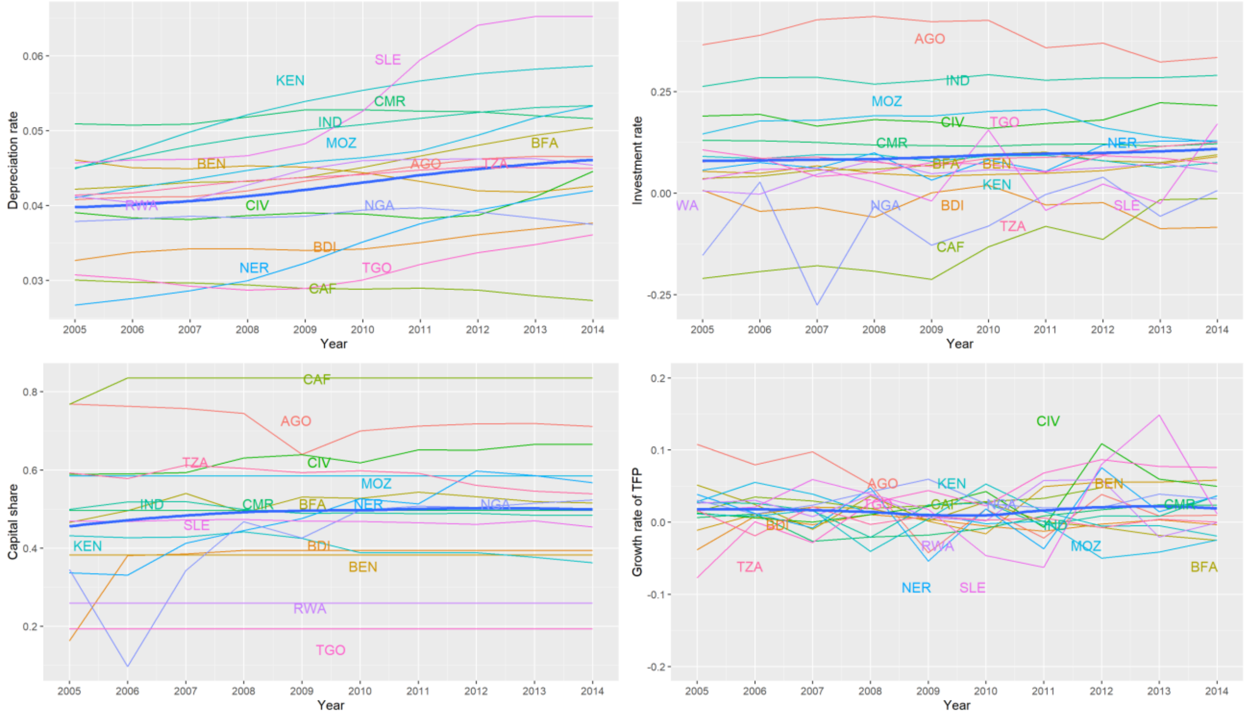

Table S4: Variance-covariance matrix of the four macroeconomic parameters

	α	G	s	δ
A	0.0264202339	-5.833711e-04	0.0039643466	-5.065750e-05
G	-0.0005833711	2.374032e-03	0.0004401364	1.593611e-05
S	0.0039643466	4.401364e-04	0.0164114237	3.938535e-04
Δ	-0.0000506575	1.593611e-05	0.0003938535	6.691660e-05
