# Supplementary material for: The Macroeconomic Impact of Increasing Investments in Malaria Control in 26 High Malaria Burden Countries: An Application of the Updated EPIC Model
Source: Int J Health Policy Manag. 2023 Oct 4;12:7132. doi: 10.34172/ijhpm.2023.7132 (PMC10590221; doi:10.34172/ijhpm.2023.7132)

Article title: The Macroeconomic Impact of Increasing Investments in Malaria Control in 26 High Malaria Burden Countries: An Application of the Updated EPIC Model

Journal name: International Journal of Health Policy and Management (IJHPM)

Authors' information: Edith Patouillard[1]*, Seoni Han[2], Jeremy Lauer[3], Mara Barschkett[4], Jean-Louis Arcand[5,6,7,8]

[1]Department of Health Financing and Economics, World Health Organization, Geneva, Switzerland.

[2]Korea Institute for International Economic Policy, Sejong, Korea.

[3]Strathclyde Business School, University of Strathclyde, Glasgow, UK.

[4]Federal Institute for Population Research and Department of Public Economics, German Institute of Economic Research (DIW Berlin), Berlin, Germany.

[5]Department of International Economics, The Graduate Institute, Geneva, Switzerland.

[6]Global Development Network, New Delhi, India.

[7]Mohammed VI Polytechnic University, Rabat, Morocco.

[8]Foundation for Studies and Research on International Development (FERDI), Clermont Ferrand, France.

***Correspondence to**: Edith Patouillard, Email: patouillarde@who.int

Citation: Patouillard E, Han S, Lauer J, Barschkett M, Arcand JL. The macroeconomic impact of increasing investments in malaria control in 26 high malaria burden countries: an application of the updated EPIC model. Int J Health Policy Manag. 2023;12:7132. doi:10.34172/ijhpm.2023.7132

Supplementary file 3. Results

Figure S2 Total investment needs (net of donor funding) under the Sustain scenario and incremental needs under the Scale-up scenario (constant 2014 US$) 2

Table S5 Percentage gains in total projected GDP for all 26 countries between 2016 and 2030, (base-case analysis) 3

Table S6 Total GDP gain (billion 2014 US$ and %) and relative contribution (absolute and percent) to GDP gain from mortality and morbidity (base-case analysis) 4

Table S7 Percentage gains in total projected GDP for all 26 countries between 2016 and 2030 (sensitivity analysis on morbidity transfer rates) 5

Table S8. Percentage gains in total projected GDP for all 26 countries between 2016 and 2030, assuming 50% of incremental investment needs is paid out by domestic savings 6

Table S9 Percentage gains in total projected GDP for all 26 countries between 2016 and 2030, assuming 90% of incremental investment needs is paid out by domestic savings 6

Figure S3 Gains in projected GDP in billions US$ 2014 between 2016 and 2030, by country income group. 7

Figure S4 Gains in projected GDP in percentage between 2016 and 2030, by country income group. 7

Figure S5 Percentage share of estimated macroeconomic benefits attributed to averted mortality and averted morbidity across all 26 countries in 2020, 2025 and 2030 8

Figure S2 Total investment needs (net of donor funding) under the Sustain scenario and incremental needs under the Scale-up scenario (constant 2014 US$)

Figure S2 displays trends in total investment needs, net of donor funding under the Sustain scenario and in incremental needs under the Scale-up scenario.

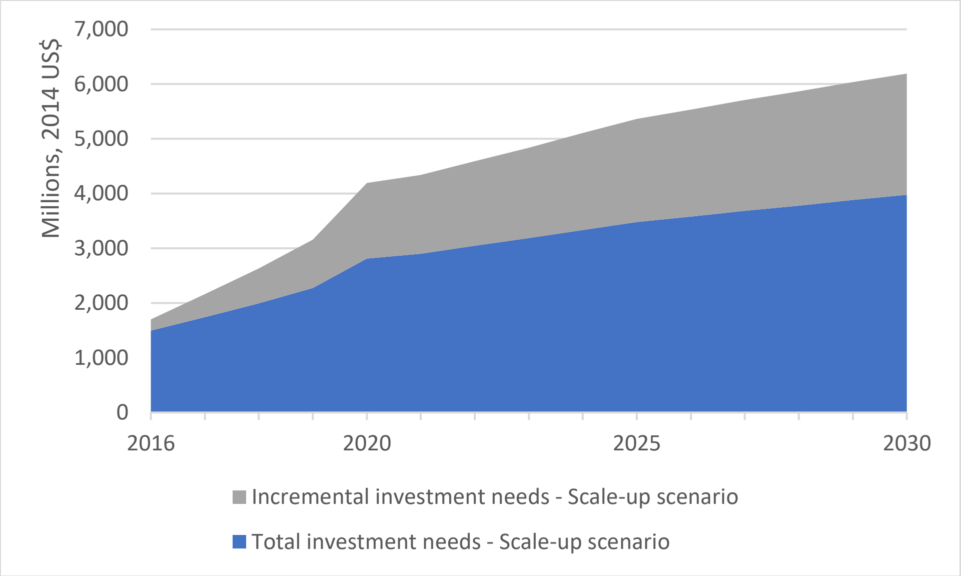

Table S5 Percentage gains in total projected GDP for all 26 countries between 2016 and 2030, (base-case analysis)

Year	GDP percent gain (mean)	GDP percent gain (95%UI, lower value)	GDP percent gain (95%UI, upper value)
2016	0,02504	0,02497	0,02512
2017	0,05380	0,05378	0,05382
2018	0,08566	0,08561	0,08570
2019	0,11141	0,11136	0,11146
2020	0,15355	0,15347	0,15364
2021	0,17672	0,17662	0,17682
2022	0,18195	0,18187	0,18204
2023	0,18519	0,18511	0,18526
2024	0,18912	0,18906	0,18919
2025	0,19484	0,19479	0,19490
2026	0,20116	0,20111	0,20122
2027	0,20601	0,20596	0,20606
2028	0,21387	0,21382	0,21392
2029	0,22232	0,22227	0,22238
2030	0,23305	0,23297	0,23312
**2016-2023**	**0,17498**	**0,17495**	**0,17500**

Table S6 Total GDP gain (billion 2014 US$ and %) and relative contribution (absolute and percent) to GDP gain from mortality and morbidity (base-case analysis)

Year	Total GDP gain (billion US$)	GDP gain from mortality only (billion US$)	GDP gain from morbidity only (billion US$)	GDP gain from mortality (%)	GDP gain from morbidity (%)
2016	0,9587	0,0132	0,9456	1,3763	98,6237
2017	2,1830	0,0347	2,1483	1,5918	98,4082
2018	3,6816	0,0635	3,6180	1,7261	98,2739
2019	5,0709	0,0895	4,9814	1,7647	98,2353
2020	7,3954	0,1205	7,2748	1,6298	98,3702
2021	8,9982	0,1572	8,8410	1,7474	98,2526
2022	9,7875	0,2033	9,5843	2,0768	97,9232
2023	10,5232	0,2712	10,2520	2,5769	97,4231
2024	11,3443	0,3590	10,9854	3,1643	96,8357
2025	12,3337	0,4800	11,8538	3,8915	96,1085
2026	13,4239	0,6227	12,8011	4,6391	95,3609
2027	14,4881	0,7747	13,7134	5,3473	94,6527
2028	15,8409	0,9382	14,9027	5,9224	94,0776
2029	17,3379	1,2557	16,0822	7,2426	92,7574
2030	19,1280	1,7665	17,3615	9,2351	90,7649
**2016-2030**	**152,4954**	**7,1500**	**145,3455**	**4,6886**	**95,3114**

Table S7 Percentage gains in total projected GDP for all 26 countries between 2016 and 2030 (sensitivity analysis on morbidity transfer rates)

Year	GDP percent gain (mean)	GDP percent gain (95%UI, lower value)	GDP percent gain (95%UI, upper value)
2016	0,01991	0,01986	0,01997
2017	0,04318	0,04316	0,04319
2018	0,06927	0,06924	0,06930
2019	0,09050	0,09046	0,09054
2020	0,12448	0,12442	0,12454
2021	0,14342	0,14335	0,14349
2022	0,14818	0,14813	0,14823
2023	0,15130	0,15126	0,15134
2024	0,15495	0,15492	0,15498
2025	0,16019	0,16017	0,16021
2026	0,16582	0,16581	0,16583
2027	0,17025	0,17024	0,17025
2028	0,17718	0,17717	0,17718
2029	0,18489	0,18488	0,18489
2030	0,19482	0,19480	0,19484
**2016-2030**	**0,14398**	**0,14394**	**0,14402**

Table S8. Percentage gains in total projected GDP for all 26 countries between 2016 and 2030, assuming 50% of incremental investment needs is paid out by domestic savings

Year	GDP percent gain (mean)	GDP percent gain (95%UI, lower value)	GDP percent gain (95%UI, upper value)
2016	0,02505	0,02497	0,02512
2017	0,05116	0,05114	0,05118
2018	0,07953	0,07950	0,07957
2019	0,10101	0,10098	0,10105
2020	0,13703	0,13698	0,13709
2021	0,15459	0,15454	0,15463
2022	0,15444	0,15444	0,15445
2023	0,15253	0,15249	0,15257
2024	0,15153	0,15145	0,15161
2025	0,15252	0,15240	0,15264
2026	0,15447	0,15432	0,15463
2027	0,15528	0,15509	0,15547
2028	0,15944	0,15921	0,15966
2029	0,16446	0,16421	0,16471
2030	0,17204	0,17177	0,17232
**2016-2030**	**0,13910**	**0,13894**	**0,13925**

Table S9 Percentage gains in total projected GDP for all 26 countries between 2016 and 2030, assuming 90% of incremental investment needs is paid out by domestic savings

Year	GDP percent gain (mean)	GDP percent gain (95%UI lower value)	GDP percent gain (95%UI, upper value)
2016	0,02505	0,02497	0,02512
2017	0,04852	0,04850	0,04854
2018	0,07340	0,07337	0,07343
2019	0,09061	0,09059	0,09063
2020	0,12050	0,12048	0,12051
2021	0,13242	0,13241	0,13243
2022	0,12689	0,12681	0,12697
2023	0,11982	0,11967	0,11997
2024	0,11387	0,11364	0,11409
2025	0,11010	0,10981	0,11040
2026	0,10768	0,10731	0,10804
2027	0,10443	0,10399	0,10487
2028	0,10486	0,10436	0,10537
2029	0,10644	0,10588	0,10700
2030	0,11087	0,11025	0,11148
**2016-2030**	**0,10313**	**0,10285**	**0,10342**

Figure S3 Gains in projected GDP in billions US$ 2014 between 2016 and 2030, by country income group. Note: results for the nine lower-middle income country.

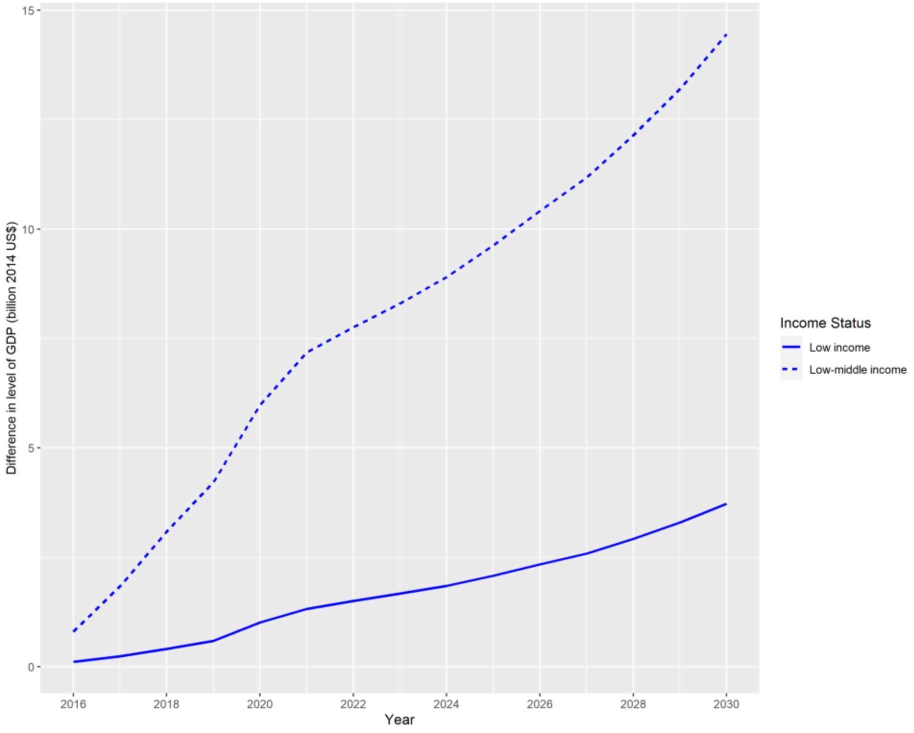

Figure S4 Gains in projected GDP in percentage between 2016 and 2030, by country income group. Note: results for the nine lower-middle income country.

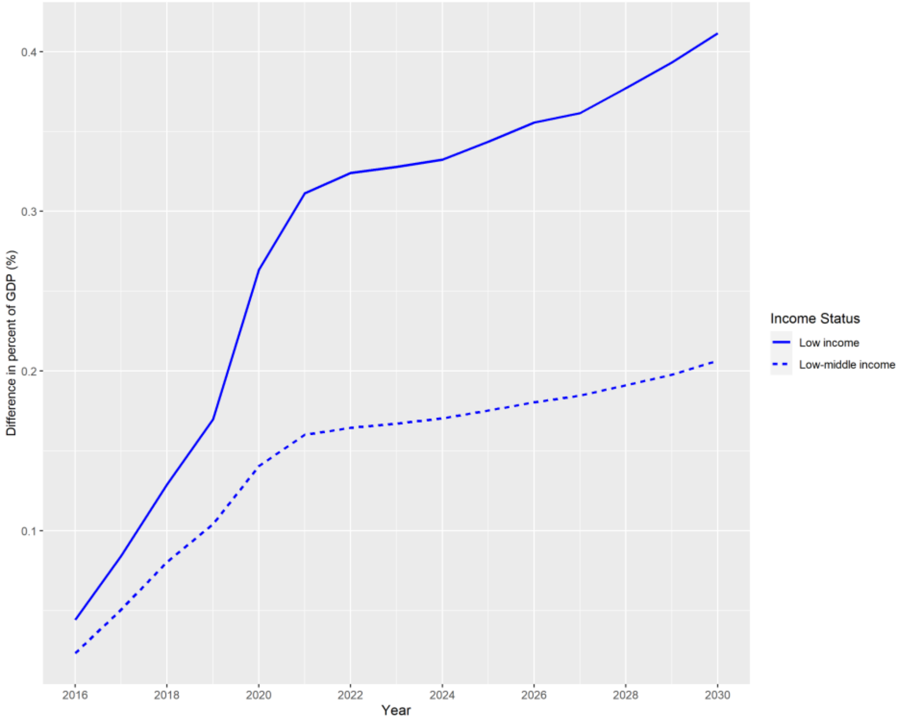

Figure S5 Percentage share of estimated macroeconomic benefits attributed to averted mortality and averted morbidity across all 26 countries in 2020, 2025 and 2030

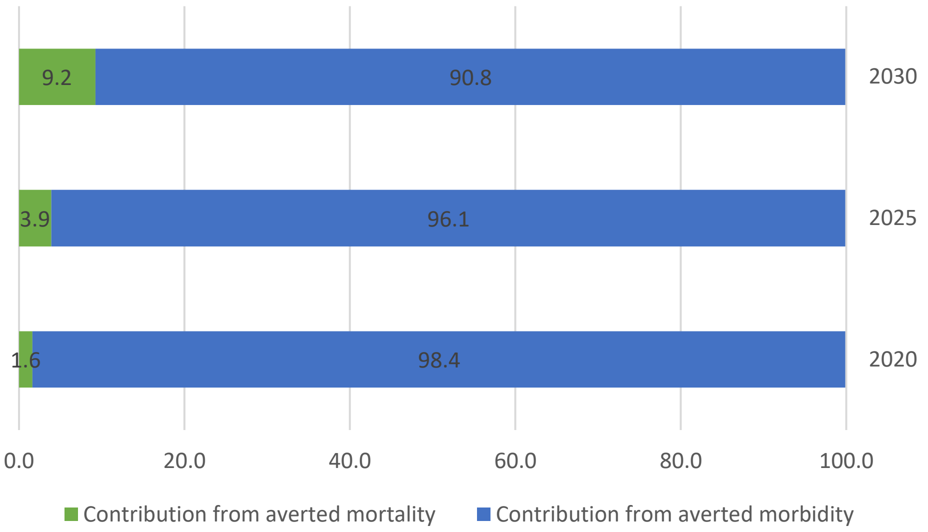

Supplement: Supplementary file 3 — Results. [file ijhpm-12-7132-s003.pdf]
